# The lncRNAs Gas5, MALAT1 and SNHG8 as diagnostic biomarkers for epithelial malignant pleural mesothelioma in Egyptian patients

**DOI:** 10.1038/s41598-024-55083-9

**Published:** 2024-02-27

**Authors:** Dina Mohamed Elkahwagy, Caroline Joseph Kiriacos, Mohamed Emam Sobeih, Ola M. Reda Khorshid, Manar Mansour

**Affiliations:** 1 Pharmaceutical Biology and Microbiology Department, Faculty of Pharmacy and Biotechnology, German University in Cairo, Cairo, Egypt; 2 Pharmaceutical Biology Department, Faculty of Pharmacy and Biotechnology, German University in Cairo, Cairo, 11835 Egypt; 3 Department of Medical Oncology, National Cancer Institute, NCI, Cairo University, Cairo, Egypt; 4 Department of Pharmaceutical Biology, Faculty of Pharmacy and Biotechnology, German University in Cairo, Cairo, 11835 Egypt

**Keywords:** Mesothelioma, lncRNA, Biomarker, Circulating, Blood, Plasma, qPCR, Early detection, Cancer, Marker, Screening, Biological sciences, Cancer, Cancer screening

## Abstract

Long noncoding RNAs have been shown to be involved in a myriad of physiological and pathological pathways. To date, malignant pleural mesothelioma (MPM) is considered an extremely aggressive cancer. One reason for this is the late diagnosis of the disease, which can occur within 30–40 years of asbestos exposure. There is an immense need for the development of new, sensitive, inexpensive and easy methods for the early detection of this disease other than invasive methods such as biopsy. The aim of this study was to determine the expression of circulating lncRNAs in mesothelioma patient plasma to identify potential biomarkers. Ten previously identified lncRNAs that were shown to be aberrantly expressed in mesothelioma tissues were selected as candidates for subsequent validation. The expression of the ten selected candidate lncRNAs was verified via quantitative PCR (qPCR) in human plasma samples from mesothelioma patients versus healthy controls. The expression levels of circulating GAS5, SNHG8 and MALAT1 were significantly greater in plasma samples from patients than in those from controls. The ROC analysis of both MALAT1 and SNHG8 revealed 88.89% sensitivity and 66.67% specificity. The sensitivity of these markers was greater than that of GAS5 (sensitivity 72.22% and specificity 66.67%). The regression model for GAS5 was statistically significant, while that for SNHG8 and MALAT1 was not significant due to the small sample size. The area under the curve (AUC) of the three ROC curves was acceptable and significant: 0.7519 for GAS5, 0.7352 for SNHG8 and 0.7185 for MALAT1. This finding confirmed their ability to be used as markers. The three lncRNAs were not affected by age, sex or smoking status. The three lncRNAs showed great potential as independent predictive diagnostic biomarkers. Although the prediction model for MALAT1 did not significantly differ, MALAT1 was significantly expressed in patients more than in controls (p = 0.0266), and the recorded sensitivity and specificity were greater than those of GAS5.

## Introduction

Malignant mesothelioma is an aggressive rare tumor that attacks the serosal surface or the mesothelial lining of the peritoneum, pericardium, tunica vaginalis and pleura^1^. The most prevalent of these four is the pleura. More than 90% of patients have malignant pleural mesothelioma (MPM)^2,3^. Malignant pleural mesothelioma is divided into three histologic characteristics: epithelioid, sarcomatoid and biphasic. The most common subtype is epithelioid, accounting for 80% of all subtypes^4^, while the second most common subtype is sarcomatoid^5,6^.

The aggressiveness of the MPM lies mainly in the latency period of the disease. The disease is often diagnosed after 30–40 years of asbestos exposure. Asbestos exposure was shown to be the major cause of this cancer in 90% of patients^7^. Approximately 70–80% of the cases involved occupational exposure^8^. Consequently, patients are diagnosed at a very late stage of the disease^9^. Furthermore, the symptoms that ultimately appear after this latent period are actually not specific to MPM, and this could add up to the disease combativeness. The symptoms are generally chest pain, dyspnea and pleural effusion^10^. This aggressiveness could be manifested in the poor prognosis of the disease as well as its survival. The median survival time is only 9–12 months, with a 5-year survival rate of only 5%^4^.

The status of the disease differs between Egypt and other regions in terms of global patterns**.** Differences include type of exposure, male:female ratios and age range^11–14^. Such variations could be explained by asbestos pollution in residential areas. Over 80% of nonoccupational MPM cases were found to be due to neighborhood exposure; MPM patients were admitted to the National Cancer Institute (NCI) between 1989 and 1999^14^.

Finally, there is a continuous risk of asbestos exposure by construction workers because there are more than 20,000 buildings containing asbestos; thus, whenever there is a need for maintenance, renovation or demolition for any building containing asbestos, there is an accompanying risk for mesothelioma^15^. Environmental exposure also carries a continued risk for mesothelioma as long as there is a continuous source of asbestos. Consequently, the number of mesothelioma patients who will appear in the near or far future may not decrease. The establishment of new non-invasive early diagnostic biomarkers is needed for patients aiming to obtain earlier treatment opportunities.

Currently, the diagnosis of MPM is invasive and expensive. Early detection of MPM before the patient becomes symptomatic via non-invasive methods is urgently needed. Recently, research has focused on identifying tumor biomarkers in blood^16^. Examples of biomarkers in blood are fibulin-3, mesothelin, osteopontin and hyaluronan. These proteins are proposed as diagnostic and prognostic biomarkers^17–20^. However, many limitations have been highlighted. Researchers are currently endeavouring to find new, fast, cheap, relatively non-invasive and easy diagnostic tools.

One such avenue is the recently discovered non coding RNA (ncRNA). Initially believed to be only transcriptional ‘debris’^21^, Recent accumulating evidence has now found that a growing number of ncRNA exert cellular functions^22^. One identified type is the long non coding RNAs (lncRNAs).

Next-generation sequencing technology has shed light on a myriad of lncRNAs that are involved in many cancerous diseases. Based on their functions, these RNAs are important for normal homeostasis, and any deviation can lead to abnormal gene regulation or peculiar biological functions and eventually threaten disease progression^23,24^. Deregulation of lncRNAs accompanies cancer onset and progression or even synchronizes different cancer hallmarks^25–30^.

A myriad of oncogenic signalling pathways are activated by their deregulation. These influential molecules can cross cell membranes and travel to body fluids^31^ despite the prevalence of ribonucleases (RNases)^32^. The primary justification behind their stability in circulation is their tendency to be carried in extracellular vesicles, with other reasoning being their stabilizing association with lipoprotein complexes, RNA-binding proteins^33^ or apoptotic bodies^34^. As a result, nucleic acids have recently been proposed as potential diagnostic markers^35^. While microRNAs (miRNAs), another form of endogenous ncRNA, have been widely examined in biomarker studies, lncRNAs have only just recently began to garner attention^36^. Many miRNAs have also been characterized in MPM^37–40^ and studies have established crosstalk between lncRNA and miRNA networks^41–43^. This implies that dysregulation in one may affect the expression of the other. One such example is the lncRNA MEG8, which was found to inhibit miR-15a-5p and miR-15b-5p of the tumor suppressor family miR-15, found to also be down-regulated in MPM^44^. This further backs the potential lncRNAs may have as markers for diseases.

LncRNAs can potentially serve as molecular biomarkers with diagnostic and prognostic value, and their potential in this area has indeed been extensively evaluated and proven in other types of cancers. One such successful application is prostate cancer associated 3 (PCA 3), a lncRNA approved by the Food and Drug Administration (FDA) and sold as Progensa by Hologic Gen Probe for the diagnosis of prostate cancer^45–47^.

Taking all these points into account, preexisting studies linked to both MPM and lncRNAs were reviewed, and 10 candidates were considered either because of their direct (i.e., reported dysregulation in biopsies of MPM patients) or indirect association (i.e., reported link to MPM through in silico analysis or cell lines), as outlined in Table 1.

**Table 1:** Summary of the lncRNAs’ information used in the study, including the references of the studies from which they were derived.

Gene Symbol	Gene ID	Relevance to MPM	References
*POT1-AS1*	ENSG00000224897	Dysregulation in MPM tissue	^48^
*LINC00689*	ENSG00000231419	In-silico analysis indicates potential use as prognostic biomarker for MPM	^49^
*ZFAS1*	ENSG00000177410	Associated with EMT transition in MPM	^50^
*CASC2*	ENSG00000177640	In region of chromosomal loss in 37% of human mesothelioma cells obtained from patients Associated with EMT transition in MPM	^50,51^
*SNHG8*	ENSG00000269893	Dysregulation in MPM tissue	^48^
*MALAT1*	ENSG00000251562	In-silico analysis indicates its overexpression and potential as biomarker for MPM Upregulated levels in mesothelioma cell lines	^50,52^
*GAS5*	ENSG00000234741	Dysregulation in MPM tissue Appropriate complementary marker in a panel of calretinin and mesothelin	^53^
*PCAT6*	ENSG00000228288	Relation of PCAT6 with lysine demethylase in all histological subtypes of MPM. Lysine demethylase is upregulated in 14% of MPM cases and induces EMT	^50,54–57^
*PVT1*	ENSG00000249859	Upregulated levels were associated with lower cisplatin sensitivity Dysregulation in mesothelioma cell lines	^58,59^
*H19*	ENSG00000130600	In-silico analysis indicates its overexpression and potential as biomarker for MPM	^50^

Thus, this study aimed to investigate the potential of using the following ten candidate lncRNAs as diagnostic biomarkers (GAS5, MALAT1, PCAT6, PVT1, H19, ZFAS1, SNHG8, CASC2, POT1-AS1 and LINC00689) in the plasma of MPM patients.

## Materials and methods

### Study population

This study was carried out between September 2021 and June 2023 at German University in Cairo (GUC). A total of 18 patients newly diagnosed with untreated epithelioid MPM were recruited from the NCI. Fifteen control samples were also obtained from healthy volunteers who did not have any history of malignancies. Patients who received any type of treatment or were diagnosed with any subtype other than epithelioid were excluded. Written informed consent was obtained from each individual participant, and the experimental protocol was approved by the Clinical Research Ethics Committee of the NCI and GUC. Also all experiments were performed in accordance with relevant guidelines and regulations of the Declaration of Helsinki. The characteristics of the patients in the study group are presented in Table 2.

**Table 2:** Characteristics of the study groups.

Parameter	Patients (n = 18)	Controls (n = 15)	p value (< 0.05)
Age; mean ± SD	61.78 ± 8.647	63.87 ± 8.684	0.4955
Gender n,%			
Males	12, (66.67%)	10, (66.67%)	> 0.9999
Females	6, (33.33%)	5, (33.33%)	
Smoking status n,%			
Smoker	8, (44.44%)	4, (26.67%)	0.4688
Non smoker	10, (55.56%)	11, (73.33%)	
Histology n,%			
Epithelioid	18, (100%)	–	
Sarcomatoid	0	–	–
Biphasic	0	–	

### Sample collection and preparation

Peripheral blood was collected in EDTA K3 tubes (Chongqing New World Trading Co., Ltd., China). Blood samples were centrifuged at 2000 × g for 20 min at 4 °C. Visual hemolysis assessment was performed using a hemolysis chart. Afterwards, the plasma was separated and stored at − 80 °C until use.

### RNA isolation and expression analysis

Total RNA from plasma was extracted using the miRNeasy Serum/Plasma Kit (Qiagen, Germany) according to the manufacturer’s instructions. cDNA was synthesized using a QuantiTect Rev. Transcription Kit (Qiagen, Germany). Subsequent qPCR was carried out with StepOne® Real-Time PCR (Applied Biosystems, USA). TaqMan™ Gene Expression Assays (Thermo Scientific, USA) were chosen for the amplification and quantification of the lncRNAs. The assay IDs of the probe-based assays are listed in Additional file 1. The prevalently used gene GAPDH was chosen for housekeeping gene normalization because of its stable expression in circulation and because its use was in accordance with Livak’s method for calculating fold change^60^. Altered expression was considered to indicate a fold change < 0.5 or > 2.0^61^. The values of ΔCt, ΔΔCt and relative quantification (RQ) were subsequently calculated.

### Statistical analyses

All the statistical analyses were performed using GraphPad Prism version 8.0.1.

A minimum sample size of 14 was calculated using G-power calculations using an effect size derived from a previous study published with a similar aim and target population^62^, 1-β (power) of 0.8, and an α (i.e., accepted type-I error) of 0.05, reported as per the “Reporting Recommendations for Tumor Marker Prognostic Studies (REMARK)”: An Abridged Explanation and Elaboration” guidelines^63^.

Numerical data are presented as the means (S.E.M.) and standard deviations (S.D.) or medians and ranges, as appropriate, while categorical data are presented as numbers and percentages. For categorical data, comparisons between two groups were performed with the chi-square test or Fisher’s exact test, as appropriate. Numerical data were tested for normality using the Shapiro‒Wilk and Kolmogorov tests. Student’s t test and the Mann‒Whitney U test were used to evaluate differences between patients and controls. The classification of lncRNAs was performed by receiver operating characteristic (ROC) curve analysis. The accuracy of the marker was determined by the area under the curve (AUC) with a 95% confidence interval (95% CI). Univariate logistic regression was performed for each lncRNA separately, with the dependent variable being the dichotomous disease status and the independent variable being GAS5, MALAT1 or SNHG8. The values assessed were the coefficient of the logistic regression equation, represented as an odds ratio. Optimal cut-offs were determined as those that maximize both sensitivity and specificity through the Youden index^64^. In all the cases, p values less than 0.05 were considered to indicate statistical significance.

### Ethics approval and consent to participate

All participants with mesothelioma were selected from the National Cancer Institute Hospital from April 2022–January 2023 during the diagnosis committee for lung related malignancies conducted on Sunday every week. The healthy controls were either recruited from outpatient clinics or were relatives of the patients. A total of 33 individuals were recruited for the study; 18 were newly diagnosed MPM patients, and 15 were healthy controls. This study was approved by the German University in Cairo and National Cancer Institute Ethics Committee. Moreover, informed consent for participation and publication of potential results was obtained from all patients before recruitment into the study.

## Results

To the best of our ability, REMARK guidelines were followed in data interpretation, analysis and presentation.

### Candidate LncRNA expression in MPM plasma samples

A literature search revealed 10 candidate lncRNAs, namely, PVT1, H19, PCAT6, ZFAS1, CASC2, POT1-AS1, GAS5, SNHG8, MALAT1 and LINC00689. LncRNAs’ detectability in plasma of 18 MPM patients were measured in comparison to controls. GAPDH was chosen as a reference candidate for normalization of lnRNAs. Three of the ten lncRNAs, GAS5, SNHG8 and MALAT1 were detected in the plasma samples of all MPM patients and healthy controls. However, PVT1, H19, PCAT6, ZFAS1, CASC2, POT1-AS1 and LINC00689 were either not stably expressed in any of the samples or were not detectable.

### Circulating GAS5, SNHG8 and MALAT1 as diagnostic markers for MPM

The three lncRNAs GAS5, SNHG8 and MALAT1 exhibited stable expression and were consistently upregulated in patients compared to controls. The normality of the data from each set of patients and controls was tested by the Shapiro‒Wilk and Kolmogorov–Sminrov tests. Both arms were normally distributed for GAS5, while for SNHG8 and MALAT1, the data were not normally distributed.

**Figure 1:**
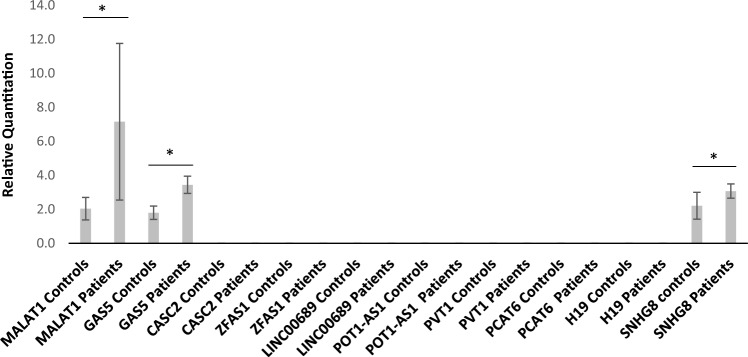
Relative quantification (RQ) of MALAT1, GAS5 and SNHG8 expression compared to the rest of undetermined lncRNASs. Only MALAT1, GAS5 and SNHG8 were detected in the plasma of both groups. All three showed consistent upregulation in plasma of patients more than controls. The 2^−ΔΔCt^ method was used to calculate RQ as representation for expression levels. The RQ for MALAT1 was 7.16 in patients and 2.04 in controls. For GAS5, the RQ in patients was 3.44, while in controls 1.80. The SNHG8 RQs for patients and controls were 3.07 and 2.21, respectively. Statistical analysis of each expressed lncRNA was performed. All three were found to be statistically significant. *Indicates p < 0.05. Unpaired Student’s t test was used for parametric data, while Mann‒Whitney U test was used for nonparametric data. Results were presented as the mean ± SEM.

An unpaired Student’s t test was performed for the GAS5 data. A statistically significant change was found between the two groups, with a p value = 0.013 and a 95% confidence interval of 0.3718–2.918. SNHG8 and MALAT1 were tested using the Mann‒Whitney U test, with significant differences found between patients and controls, with p values = 0.021 and 0.032, respectively. All three were found to be upregulated. Figure 1 shows the RQs of all the lncRNAs in the plasma of patients with malignant pleural mesothelioma relative to those in healthy controls, representing the fold change in expression.

MALAT1 exhibited the greatest upregulation at a fold change of 7.16, followed by 3.44 for GAS5 and 3.07 for SNHG8. Circulating GAS5, SNHG8 and MALAT1 levels were not affected by age, sex or smoking status, as determined by significance tests and chi-square tests/Fisher’s exact tests where appropriate.

### ROC

**Figure 2:**
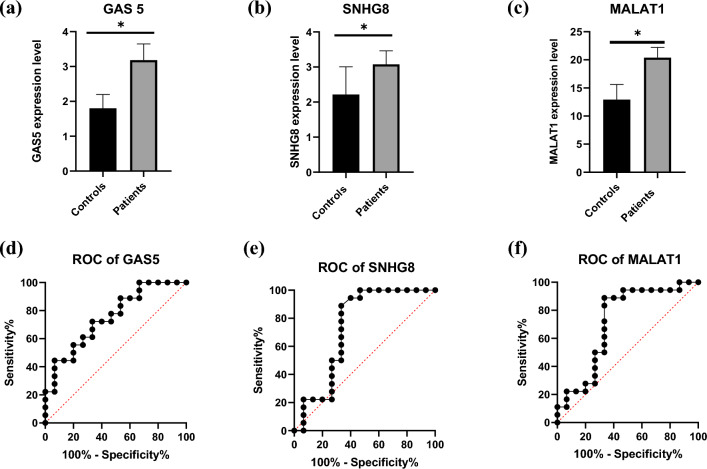
ROC curve of lncRNA expression levels and their subsequent diagnostic power. Results of Student’s test, Mann–Whitney U test used to analyse the difference between the patients and control groups (**a**) Unpaired Student’s t test was performed for GAS5 data. (**b**) SNHG8 and (**c**) MALAT1 data were analysed by Mann‒Whitney U test. All three were found to be statistically significant. *Indicates p < 0.05. The diagnostic power of the three lncRNAs was determined by ROC curve analysis. The three achieved an acceptable AUC. (**d**) The ROC curve of GAS5 had an AUC of 0.7519 (p value = 0.0140, 95% CI = 0.5864–0.9173). The sensitivity and specificity of GAS5 were 72.22% and 66.67%, respectively. (**e**) The discriminative power of SNHG8 was measured, and the AUC was 0.7352 (p value = 0.0217, 95% CI = 0.5401–0.9303). (**f**) The AUC of MALAT1 was 0.7185 (p value = 0.0329, 95% CI = 0.5279–0.9091). The sensitivity and specificity of SNHG8 and MALAT1 were 88.89% and 66.67%, respectively.

The diagnostic power of the three lncRNAs was first determined by receiver operating characteristic (ROC) curve analysis. The AUC of GAS5 was 0.7519 (p value = 0.014, 95% CI = 0.586–0.917), with 72.22% sensitivity and 66.67% specificity, with an optimum cut-off > 1.997. SNHG8 had an AUC of 0.735 (p value = 0.022, 95% CI = 0.540–0.930) and was at an optimum cut-off of 1.465. The sensitivity and specificity of SNHG8 were 88.89% and 66.67%, respectively. The optimum cut-off for MALAT1 expression was > 1.346, for a sensitivity of 88.89% and a specificity of 66.67%, with an AUC of 0.719 (p value = 0.033, 95% CI = 0.528–0.909). The plasma expression of each lncRNA in patients and controls, as well as the ROC curve for each, are shown in Fig. 2.

### Univariate logistic regression

Logistic regression analysis was performed to evaluate the influence of GAS5 expression on the outcome of diagnosis as another metric of diagnostic performance. The coefficients of the logistic regression indicate the levels of association, represented as odds ratios, with values higher than 1 indicating a direct correlation between the biomarker and diagnosis, while values less than 1 indicate an inverse relationship.

The odds ratio of GAS5 was found to be 1.61, indicating that an increase in the independent variable GAS5 by one unit will increase the probability of being “diseased” by 1.61. With MALAT1, the odds ratio was also found to be directly proportional at 1.13. Although the differences in the expression of both of these lncRNAs were statistically significant, SNHG8 was not significantly related to the expression of these lncRNAs, with an odds ratio of 1.81. This could be attributed to the variability in expression levels between patients, as when one outlier was excluded, statistical significance was achieved (data not shown).

## Discussion

LncRNA expression has been found to be dysregulated in a multitude of cancers, and as the number of lncRNAs is almost double that of protein-coding genes^65,66^, these biomolecules are likely major participants in the molecular pathogenesis of these cancers. Thus, extensive investigations have been directed toward the potential of lncRNAs as biomarkers for cancer detection owing to the abovementioned points.

The REMARK guidelines were used as the framework for reporting the study’s results in establishing and validating a biological link between MPM and potential lncRNA biomarkers in the plasma of a patient cohort^63,67^.

Ten lncRNAs were chosen based on their previously reported altered expression in MPM. The potential of these markers as diagnostic markers for MPM from liquid biopsies was evaluated. The expression of MALAT1, GAS5, H19, PCAT6, PVT1, SNHG8, POT1-AS1, ZFAS1, CASC2 and LINC00689 was measured. Only MALAT1, GAS5 and SNHG8 were consistently detected in the plasma of all the patient and control samples by real-time PCR. Hence, they became the focus of our study. Possible reasons for not detecting the rest on lncRNAs could be that circulating lncRNA exist in various forms, which may not all be captured by the extraction kit (for example, certain kits are targeted at enriching exosome encapsulated RNA). Some lncRNAs are also more stable than others, (Clark et al.^68^) identified approx. 240 unstable lncRNA transcripts with a half-life below 2 h in an analysis done on a mouse neuroblastoma cell-line^68^.

The impact of influencing factors such as age, sex and smoking status was statistically analysed, and the results showed the lack of influence of those three factors on the plasma levels of GAS5, MALAT1 and SNHG8. Accordingly, the expression of these genes significantly increased in the plasma of MPM patients compared to that in the plasma of controls (3.44, 7.16 and 3.07).

The best cut-off was determined by obtaining a cut-off closer to the upper left-hand corner of the curve (sensitivity/specificity balance). Given that the aim of this study was to detect and identify cancer, this method was adopted here.

Accordingly, sensitivity and specificity were evaluated by receiver operating characteristic (ROC) curves to determine the optimum balance and diagnostic power of the AUC. GAS5, SNHG8 and MALAT1 had values between 0.7 and 0.8, which are generally considered to be within the acceptable range of AUC values. The three lncRNAs had equivalent specificity values, while the sensitivity of GAS5 was lower than that of SNHG8 and MALAT1. Each marker was then evaluated independently in a model by logistic regression. The chi-square test was used to determine whether the model performed better (in terms of predicting diagnosis) when the biomarker expression values were added. The overall model was significant for both GAS5 and SNHG8, but MALAT1 did not significantly differ despite its consistent upregulation and discrimination ability. This could be linked to the wide variation in their values irrespective of their overexpression in patients, possibly because of the various isoforms expressed but not uniformly measured, or the possible effect of other confounders. Thus, it is still possible that MALAT1 can serve as a biomarker; however, a larger sample is needed that also includes other covariates that may influence MALAT1 expression.

Our GAS5 results are in agreement with those of a previous study^69^ in which the expression levels of GAS5 were analysed in MPM biopsy tissue, which revealed higher expression despite paradoxical downregulation of GAS5 in MPM cell lines^53^. An explanation of their presence in circulation could be that they are a result of tumor microenvironment secretions attempting to exert control on other genes^70^. Another study supported our findings, suggesting that circulating GAS5 could be a prognostic marker before and after chemotherapy in mesothelioma patients^71^. Accordingly, in combination with other potential findings, we used GAS5 as both a positive control and a biomarker in this study via a diagnostic panel.

Along with POT1-AS1, SNHG8 is a lncRNA that was investigated in MPM tissues from patients with EPP (extrapleural pneumonectomy) and in cryopreserved benign pleura tissues^48^. RT‒qPCR was used in this investigation and revealed that the level of SNHG8 was five-fold greater in MPM than in benign pleura. This was followed by an ROC curve that demonstrated the ability of SNHG8 to differentiate between benign pleura and MPM with a high degree of accuracy. Consequently, it was added into this study. Although it was found to be both stably expressed and upregulated, the overall discriminatory ability of its expression in tissue surpassed that of its circulatory expression. Nonetheless, it still holds potential as a complementary marker and a less invasive alternative.

While each marker has shown potential independently, the results still need to be validated in a larger sample size. The specificity and sensitivity of each lncRNA could also be investigated with respect to other lncRNAs, and whether each lncRNA works well could be assessed with a combined panel. Therefore, multiple regression analysis was performed with various combinations of lncRNAs, but due to the limited sample size, the data were not significantly different (data not shown).

It should be noted that the MPM patients in this study were recently diagnosed, albeit at late stages of tumor development, as is often the case due to the latent nature of the disease and its nonspecific symptoms. Using prediagnostic samples could lead to better results regarding marker panel performance, but this still needs to be verified^72^.

In summary, many published studies have already shown that lncRNAs are more sensitive than already established panels of the proteins calretinin and mesothelin with senstivity (46%). The diagnostic performance of these lncRNAs could also be improved by combining them with other biomolecules. For instance, the combination of GAS5 with calretinin and mesothelin improved the effectiveness of the existing panel of mesothelioma markers^69^. A potential future direction would be to also measure whether SNHG8 and MALAT1 could complement and potentiate preexisting biomarker panels.

## Conclusion

Ten lncRNAs were identified based on a literature screening for analysis of their potential as predictive diagnostic markers in liquid biopsies. Notably, GAS5, SNHG8 and MALAT1 were detected and further analysed as potential biomarkers. The three lncRNAs showed both dysregulation and potential for discriminating between patients and controls. The results of this study need to be validated with a larger sample size to ascertain the true performance of the lncRNAs as diagnostic biomarkers. Moreover, their use in the early detection of MPM remains to be explored.

### Supplementary Information

Supplementary Information.

## Data Availability

The datasets generated during and/or analysed during the current study are available from the corresponding author upon reasonable request.

## Abbreviations

AUC: Area under the curve
CI: Confidence interval
Ct: Cycle threshold
EPP: Extrapleural pneumonectomy
FDA: Food and Drug Administration
GUC: German University in Cairo
LncRNA: Long non coding RNA
miRNA: MicroRNA
MPM: Malignant Pleural Mesothelioma
MRI: Magnetic Resonance Imagining
NCI: National Cancer Institute
NcRNA: Non coding RNA
NPV: Negative predictive value
NTC: No-template control
PCA3: Prostate cancer associated 3
PPV: Positive predictive value
qPCR: Quantitative Polymerase Chain Reaction
REMARK: Reporting Recommendations for Tumor Marker Prognostic Studies
RNases: Ribonucleases
ROC: Receiver operating characteristics curve
RQ: Relative quantification
SD: Standard deviation
TPR: True positive rate
VATS: Video-assisted thoracoscopy
WHO: World Health Organization
