# Supplementary material for: The lncRNAs Gas5, MALAT1 and SNHG8 as diagnostic biomarkers for epithelial malignant pleural mesothelioma in Egyptian patients
Source: Sci Rep. 2024 Feb 27;14:4823. doi: 10.1038/s41598-024-55083-9 (PMC10899637; doi:10.1038/s41598-024-55083-9)
Supplement: Supplementary file 1 — Supplementary Information. [file 41598_2024_55083_MOESM1_ESM.docx]

| **TaqMan® Gene Expression Assays:** | **Assay ID** |
| --- | --- |
| GAPDH | Hs02758991_g1 |
| GAS5 | Hs05021116_g1 |
| MALAT1 | Hs00273907_s1 |
| PCAT6 | Hs01054758_g1 |
| PVT1 | Hs00413039_m1 |
| H19 | Hs00399294_g1 |
| POT1-AS1 | Hs01550335_m1 |
| LINC00689 | Hs04989865_m1 |
| ZFAS1 | Hs01379985_m1 |
| CASC2 | Hs00289594_m1 |
| SNHG8 | Hs03461282_g1 |
